# Economic evaluation of the air pollution effect on public health in China’s 74 cities

**DOI:** 10.1186/s40064-016-2024-9

**Published:** 2016-04-01

**Authors:** Li Li, Yalin Lei, Dongyan Pan, Chen Yu, Chunyan Si

**Affiliations:** School of Humanities and Economic Management, China University of Geosciences, Beijing, 100083 China; Key Laboratory of Carrying Capacity Assessment for Resource and Environment, Ministry of Land and Resources, Beijing, 100083 China; Central University of Finance and Economics, Beijing, 100081 China

**Keywords:** Air pollution, The public health effect, The economic loss, 74 cities, China

## Abstract

Air deterioration caused by pollution has harmed public health. The existing studies on the economic loss caused by a variety of air pollutants in multiple cities are lacking. To understand the effect of different pollutants on public health and to provide the basis of the environmental governance for governments, based on the dose–response relation and the willingness to pay, this paper used the latest available data of the inhalable particulate matter (PM_10_) and sulphur dioxide (SO_2_) from January 2015 to June 2015 in 74 cities by establishing the lowest and the highest limit scenarios. The results show that (1) in the lowest and highest limit scenario, the health-related economic loss caused by PM_10_ and SO_2_ represented 1.63 and 2.32 % of the GDP, respectively; (2) For a single city, in the lowest and the highest limit scenarios, the highest economic loss of the public health effect caused by PM_10_ and SO_2_ was observed in Chongqing; the highest economic loss of the public health effect per capita occurred in Hebei Baoding. The highest proportion of the health-related economic loss accounting for GDP was found in Hebei Xingtai. The main reason is that the terrain conditions are not conducive to the spread of air pollutants in Chongqing, Baoding and Xingtai, and the three cities are typical heavy industrial cities that are based on coal resources. Therefore, this paper proposes to improve the energy structure, use the advanced production process, reasonably control the urban population growth, and adopt the emissions trading system in order to reduce the economic loss caused by the effects of air pollution on public health.

## Background

Air pollution mainly refers to human activities or the natural processes that cause a certain substance to continuously enter the atmosphere at a sufficient concentration to endanger the health and cause environmental pollution. There are many types of air pollutants, the primary of which are total suspended particulates (TSP), inhalable particulate matter (PM_10_), fine particulate matter (PM_2.5_), SO_2_ and NOx, among others (Yu et al. 2008). After inhalation of harmful pollutions, humans may develop respiratory disease and can suffer from serious diseases, such as tracheitis, bronchitis, asthma, lung disease and lung cancer, for many years. The energy consumption structure in China is mainly based on coal resources, and the rapid growth of motor vehicles in cities results in increasingly more serious air pollution in China’s large cities; in addition, the questions regarding public health and air pollution have garnered widespread attention (Wilkinson and Smith 2007).

The main air pollutions in China are PM_10_, SO_2_ and NOx (Chen et al. 2001). In the 12th Five-Year period, China clearly proffered the target to reduce the total SO_2_ emissions by 8 % and increase the ratio of the urban air quality to achieve the second level of 8 % (Ministry of Environmental Protection of the People’s Republic of China 2011). According to an environmental analysis reported by the Asian Development Bank in 2013, the Chinese government was taking measures to control air pollution; however, of the world’s 10 most seriously polluted cities, 7 cities were in China. Of China’s 500 major cities, less than 1 % met the standards of the World Health Organization (Zhang 2012). In 2015, the World Health Organization released a report stating that at least one in eight people died of air pollution globally. Air pollution has become the world’s largest environmental health risk (Huanqiunet 2015).

Since January 1, 2013, the Ministry of Environmental Protection has monitored the air quality index of Beijing, Tianjin, Hebei, the Yangtze River Delta, the Pearl River Delta region, the municipality directly under the central government, the provincial capital cities and the cities specifically designated in the state plan, which are collectively called the 74 cities, in brief (China’s National Environmental Monitoring Centre 2013a). The concentrations of pollutants such as PM_10_ and SO_2_ have been monitored since November 2014 (China’s National Environmental Monitoring Centre 2013b). As World Bank (1997) didn’t identify the health effects of NOx and NOx wasn’t included in the dose–response relation of Ho and Jorgenson (2007), thus to quantitatively evaluate the economic loss due to the effects of air pollution on public health in China, this paper analyzes PM_10_ and SO_2_ based on the latest available data from January 2015 to June 2015, uses the method of foreign study on China’s economic loss due to air pollution effects on public health for reference (Wang and Smith 1999; Ho and Nielsen 2007) and estimates the economic loss caused by the effects of air pollution on public health in the 74 cities. An evaluation of the health-related economic loss can provide a basis for the government to develop and initiate preventative measure for controlling air pollution. At the same time, these findings can also improve the environmental protection awareness of the local government and the public.

## Literature review

In recent decades, industrialization and urbanization have experienced rapid development, which has resulted in increasing air pollution. According to the World Bank, there is a close relationship between air pollution and public health. There is a positive relation between the concentration of air pollutants and respiratory diseases, lung function loss, chronic bronchitis and premature death (World Bank SEPA 2007). The evaluation of health-related economic loss caused by air pollution has become a hot topic for scholars and institutions.

### Research progress on the economic loss regarding to public health impacts caused by air pollution

Ridker (1967) calculated the economic loss associated with different diseases which caused by air pollution in the USA in 1958 by using the human capital method. The results showed that the economic loss related to the effects on public health was 80.2 billion dollars in the USA. This study hailed the beginning of the calculation of health-related economic loss caused by air pollution.

Employing a survival analysis and the data from a 14- to 16-year mortality follow-up of 8111 adults in the six cities in the U.S., Dockery et al. (1993) estimated the associations between particulate air pollution and daily mortality rates. Their results confirmed that the mortality rate was associated with the level of air pollution. Using data from 1994 to 1995 in Hong Kong, Wong et al. (1999) determined that adverse health effects were evident at the current ambient concentrations of air pollutants. Samet et al. (2000) recognized an association between daily changes in the concentration of ambient particulate matter and the daily number of deaths (mortality) in the United States. Wong et al. (2002) used Poisson regression to estimate the associations between daily admissions and the levels of PM_10_ and SO_2_ in Hong Kong and London. The results confirmed that air pollution caused detrimental short-term health effects.

Using the collective data regarding to PM_10_ and SO_2_ from January 1999 to September 2000, Kaushik et al. (2006) assessed the ambient air quality status in the fast growing urban centres of Haryana state, India. Adopting the daily data during 2008 and 2009 in Beijing, Xu et al. (2014b) confirmed that short-term exposure to particulate air pollution was associated with increased ischemic heart disease (IHD) mortality.

In 1981, the concept, theory and method of environmental pollution economic loss assessment were put forward and discussed in the congress of the National Symposium on Environmental Economics (Xia 1998). Thereafter, the economic loss associated with environmental pollution was of interest to scholars. Gao et al. (1993) adopted the GEE (Generalized Estimation Equation) to study the relationship between TSP in Haidian District, Beijing and low air pollution. Using the two methods (ecology and time series) and the data of 1992 in Shenyang, Xu et al. (1996) determined that total mortality, chronic obstructive pulmonary disease (COPD), cardiovascular disease and pollution levels were significantly correlated. Jing and Ren (2000) conducted an epidemiological survey on adults who were older than 25 years using the multiple logistic regression analysis. The results showed that 6 types of respiratory system diseases or symptoms appeared with an increasing frequency as air pollution levels increased. Chen and Hong (2002) quantitatively evaluated the air pollution in Shanghai based on the risk evaluation method and found that the health effects caused by SO_2_ exhibited a gradually declining trend. Chen et al. (2010) evaluated the health impacts of particulate air pollution on urban populations in 113 Chinese cities, and it was estimated that the total economic cost of the health impact was approximately 341.4 billion Yuan, 87.79 % of which was attributable to premature deaths. Chen et al. (2015) employed a Poisson regression model to estimate residents’ health benefits in two scenarios: environmentally controlled scenario 1 and environmentally controlled scenario 2. Scenario 2 showed a potentially higher reduction of emissions and greater health benefits than scenario 1. Xu et al. (2014a) used the established model between PM_10_ and thermal environmental indicators to evaluate the PM_10_—related health risk in Beijing.

Certain scientific institutions also focused more on the economic loss associated with public health effects caused by air pollution. The World Health Organization estimated that the total loss globally caused by air pollution-related disease was 0.5 % (Murray and Lopea 1997) in 1997. In the same year, the World Bank systematically studied the health effects caused by air pollution in China (World Bank 1997). The U.S. Environmental Protection Agency estimated that the economic benefits of health and ecological improvement in the United States from 1990 to 2010 were as high as $6–50 trillion, most of which could be attributed to the decrease in the number of deaths caused by air pollution (U.S. EPA 1999). The World Health Organization reported that 80 % of the world’s cases of heart disease and stroke deaths were due to air pollution, and a total of 7 million people in the world died of air pollution in 2014 (Huanqiunet 2014). In 2015, the World Health Organization released data that at least 1 in every 8 people died of air pollution throughout the world. Air pollution has become the world’s largest environmental health risk (Huanqiunet 2015).

### Research progress on the method used to evaluate the economic loss associated with public health effects caused by air pollution

The previous studies regarding to the economic loss caused by the effects of air pollution on public health generally included the determination of economic loss using the contents of the environmental pollution assessment, the public health impact assessment and a choice of methods. Generally, the methods used were as follows.

1. Modified human capital method

Ridker (1967), Dockery et al. (1993), Wang et al. (2005), Jia et al. (2004), Wan et al. (2005), Han et al. (2006), Zhang et al. (2008), Shang et al. (2010), Han (2011), and Shen et al. (2014) quantitatively estimated the economic loss in different regions and obtained different results.

2. Illness cost method

Air pollution led to changes in the disposable income of people, particularly, an increase in medical expenses. Medical expenses became a recognized fact, and they also became a very heavy burden on civilians. Based on the above views, certain scholars obtained conclusions by analysing the illness costs caused by air pollution. These scholars include Chen et al. (2010), Zmirou et al. (1999), Hedley et al. (2008), Patankar and Trivedi (2011), Brandt et al. (2014), and Yan (2012).

3. Willingness to pay

Willingness to pay is an indirect evaluation method which constructs a simulated market to reveal people’s willingness to pay for certain environmental goods, in order to evaluate the value of environmental quality. Researchers included Carlsson and Martinsson (2001), Wang and John (2006), Koop and Tole (2004), Pascal et al. (2013), Yaduma et al. (2013), Ami et al. (2014), Istamto et al. (2014), Cai and Yang (2003), Peng and Tian (2003), Cai et al. (2007), Zhou et al. (2010), and Zeng et al. (2015).

### Literature summary

1. In the studies of the economic loss caused by air pollution, domestic and foreign researchers studied the qualitative relationship between and quantitative analysis of air pollution and its health effect. However, generally, previous studies were solely based on a country, a city or a type of air pollutant; the health effects of many types of air pollutants in a city of a typical city are moderate.
2. Regarding to the method for evaluating the economic loss caused by the effects of air pollution on public health, the deficiencies of the modified human capital method were that the life prediction of the society may not be reasonable, and the different choices of the discount rate would have a large impact on the evaluation results. The disadvantage of the illness cost method is that it may underestimate the illness value. Additionally, the method’s other disadvantage was that the individual may have a willingness to pay.

From the economic perspective, the willingness to pay method is the most reasonable method because it can reveal the value of all goods and utilities, and it can completely evaluate the economic values of environmental resources, which is currently being widely recognized and accepted. Thus, this paper utilizes the willing to pay method to evaluate the economic loss associated with public health effects caused by air pollution in 74 cities.

## Methods and data

### The dose–response relationship and the willingness to pay

To study the economic loss related to public health effects caused by air pollution, it is necessary to consider the types of public health effects and to establish the relation between the concentration of air pollutants and the effect on public health, which is called a dose–response. In different studies, the dose–response relationship is different. The indexes of public health effects caused by air pollution, which the World Bank put forward, included premature deaths, hospitalization and emergency caused by respiratory diseases, the number of restriction days caused by health problems related to the inhalation of particulate matter, lower respiratory tract infections, childhood asthma, asthma, chronic bronchitis, respiratory symptoms, and chest discomfort. This paper used the dose–response relationship of Ho and Jorgenson (2007) for reference and assumed that all the people in the 74 cities were exposed to the same concentrations of PM_10_ and SO_2_. The dose–response relationship is shown in the Eq. (1).

1 $$HE_{xrh} = DR_{xh} \times C_{rx} \times POP_{r}$$

where $HE_{xrh}$ is the h-th type of public health effect caused by the air pollutant x (including PM_10_ and SO_2_) in the region r. $DR_{xh}$ is the dose–response coefficient of the air pollutant x (unit: the number of the people suffer with the concentration of the air pollutant increasing by 1 μg/m^3^) and the h-th type of public health. $C_{rx}$ is the concentration of the air pollutant x in the region r. $POP_{r}$ is the number of the people in the region r. Ho and Jorgenson (2007) used the survey data of Beijing and Anqing in 1997 to estimate the economic loss caused by the health effects of Chinese residents using the willingness to pay method, the population of was were 6.53 million and 0.35 million respectively. Based on the loss value estimation of Ho and Jorgenson (2007), this paper modified it, as shown in Table 1. The total economic loss of the 74 cities is obtained by adding up all of the economic loss types relating to the health effects, as shown in the Eqs. (2) and (3):

2 $$HEV_{xrh} = V_{xh} \times HE_{xrh}$$

3 $${\text{THEV}} = \mathop \sum \limits_{r} \mathop \sum \limits_{x} \mathop \sum \limits_{h} HEV_{xrh}$$

where $HEV_{xrh}$ is the economic loss of the h-th type of public health effect caused by the air pollutant x (including PM_10_ and SO_2_) in the region r. $V_{xh}$ is the economic loss value of the h-th type of public health effect caused by the air pollutant x (including PM_10_ and SO_2_). ${\text{THEV}}$ is the total economic loss related to public health effects caused by the air pollutant. In calculating total health-related economic loss, the paper adds up eight effects of PM_10_ on public health and three effects of SO_2_ on public health together, which may appear double counting. As there is little literature of this issue, the paper hasn’t analyzed it.

**Table 1:** The dose–response relationship and the loss value estimation of the public health effects

	Coefficient of the dose–response relationship		Loss value estimation (yuan, the price in 2002)	Loss value estimation (yuan, the mean price from January 2015 to June 2015)
	The lower limit scenario	The highest limit scenario	Ho and Jorgenson’s estimation	Modified estimation
Effects of PM_10_ on public health				
Premature death	1.3	2.6	370,000	528,370.2
Hospitalization caused by respiratory disease	12	12	1751	2500.5
Emergency	235	235	142	202.8
Number of restriction days	18,400	57,500	14	20
Lower respiratory tract infections and childhood asthma	23	23	80	114.2
Asthma	1770	2608	2.5	3.57
Chronic bronchitis	61	61	48,000	68,545.33
Respiratory symptoms	49,820	183,000	3.7	5.28
Effects of SO_2_ on public health				
Premature death	1	2.6	370,000	528,370.2
Chest discomfort	10,000	10,000	6.2	8.85
Lower respiratory tract infections and childhood asthma	5	5	6.2	8.85

Source: Ho and Jorgenson (2007)

### Data

**Fig. 1:**
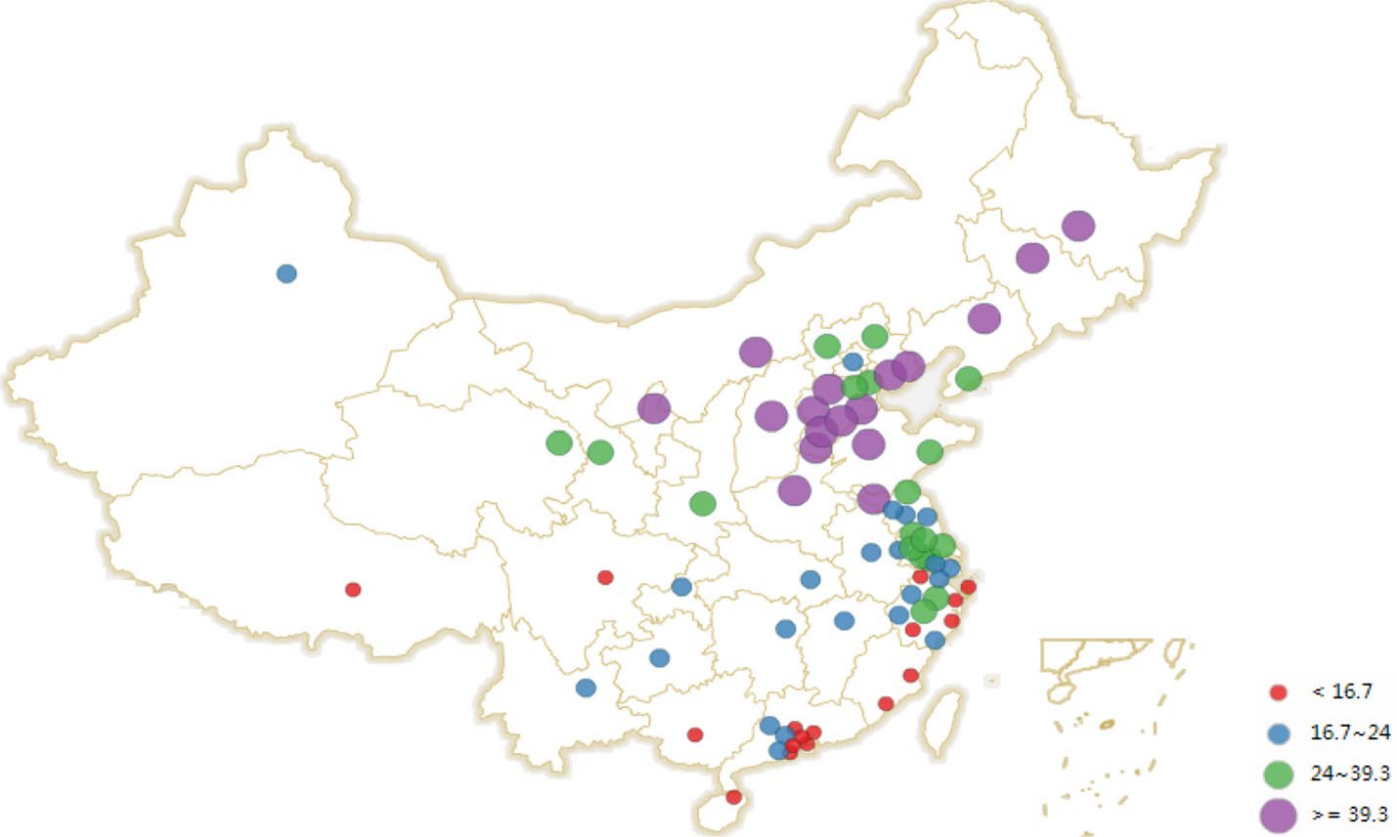
Monthly mean concentration of SO_2_ in China’s 74 cities

**Fig. 2:**
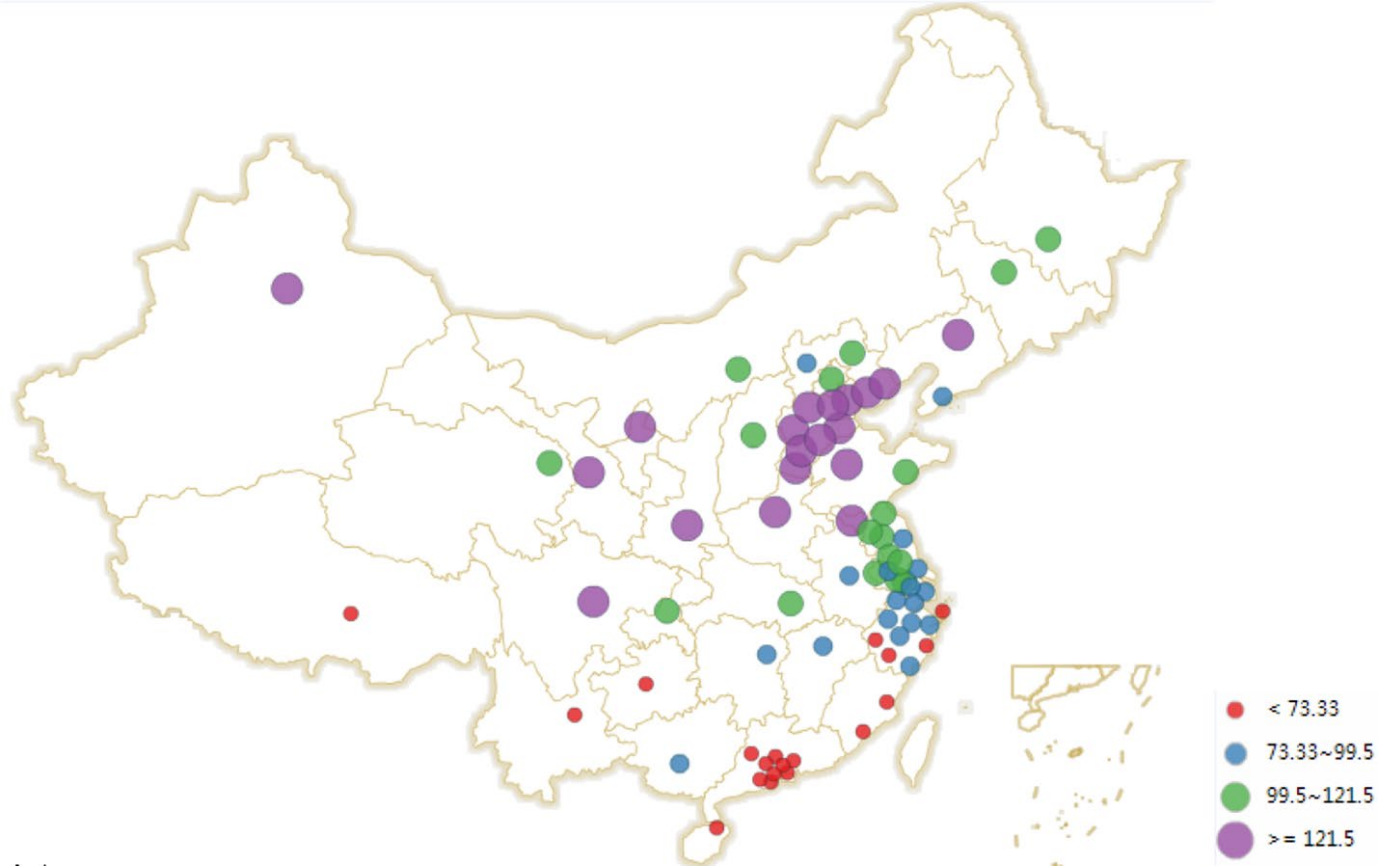
Monthly mean concentration of PM_10_ in China’s 74 cities

The environmental data of 74 cities during the period from January 2015 to June 2015 were reported by China’s National Environmental Monitoring Centre (2015a, b, c, d, e, f), which mainly contained the monthly mean concentrations of PM10 and SO_2_ (Figs. 1, 2). The number of the population and GDP in 74 cities were obtained from Askcinet (2015).

From Figs. 1 and 2, Taiyuan, Shenyang and Yinchuan are the top three cities with the highest monthly mean concentration of SO_2_. Baoding, Zhengzhou and Xingtai are the top three cities with the highest monthly mean concentration of PM_10_. According to the ambient air quality standard GB3095-2012, the paper compared Chongqing, Baoding and Xingtai with Beijing and found that: (1) For SO_2_, Beijing and Chongqing achieved the first level of national standards from January 2015 to June 2015. Baoding and Xingtai achieved the first level of national standards from April to June 2015, and they achieved the second level of national standards from January to March 2015. (2) For PM_10_, Beijing achieved the second level of national standards from January 2015 to June 2015. Chongqing achieved the second level of national standards from February 2015 to June 2015, which didn’t achieve the national standards in January 2015. Baoding and Xingtai achieved the second level of national standards from April to June 2015, but they didn’t achieve the national standards from January to March 2015. Overall, the monthly mean concentration of SO_2_ and PM_10_ in the 4 cities appeared a downward trend.

## Results and discussions

### The total economic loss associated with public health effects caused by air pollution in 74 cities

The dose–response relationship and the loss value estimation of the public health effects in different cities vary; therefore, this paper establishes different scenario parameters for the lowest limit scenario and the highest limit scenario in order to evaluate the total economic loss related to the effects of air pollution on public health in 74 cities.

1. The lowest limit scenario

As shown in Table 2, there were 84,917 premature deaths caused by PM_10_ and SO_2_. There were 646,282 hospitalizations caused by respiratory disease, 12.66 million emergencies, more than 990 million restriction days and 1.23 million lowest respiratory tract infections and occurrence of childhood asthma due to PM_10_. However, the economic loss caused by the effects of SO_2_ on public health was less than that of PM_10_.

**Table 2:** The total lowest economic loss caused by the effects of air pollutants on public health in 74 cities

	Coefficient of the dose–response relation	Cases	Loss value estimation (yuan, the mean price from January 2015 to June 2015)	Economic loss (million yuan)
Effects of PM_10_ on public health				
Premature death	1.3	70,014	528,370.2	36,993.31
Hospitalization caused by respiratory disease	12	646,282	2500.5	1616.03
Emergency	235	12,656,359	202.8	2566.71
Number of restriction days	18,400	990,965,979	20	19,819.32
The lowest respiratory tract infections and childhood asthma	23	1,238,707	114.2	141.46
Asthma	1770	95,326,619	3.57	340.32
Chronic bronchitis	61	3,285,268	68,545.33	225,189.78
Respiratory symptoms	49,820	2,683,148,101	5.28	14,167.02
Total economic loss of the public health effect caused by PM_10_	300,833.95			
Effects of SO_2_ on public health				
Premature death	1	14,903	528,370.2	7874.30
Chest discomfort	10,000	149,030,000	8.85	1318.92
The lowest respiratory tract infections and childhood asthma	5	74,515	8.85	0.66
Total economic loss caused by the effects of SO_2_ on public health effect	9193.88			
Total economic loss related to public health effects	310,027.82			
Economic loss related to public health effects per capita	598 yuan			
GDP in the 74 cities	18,987,854			
Total economic loss of the public health effects accounting for the GDP	1.63 %			

This paper calculated that the total health-related economic loss caused by the air pollutant in 74 cities was approximately 310 billion yuan, explaining approximately 1.63 % of the 74 cities’ GDP, which was higher than the result of Wei et al. (2012). The total economic loss of the public health effect caused by PM_10_ was 300.8 billion yuan, explaining approximately 97.03 % of the total economic loss, which was the major economic loss and was consistent with the result of Zhang (2012). The economic loss caused by chronic bronchitis, which was approximately 225.2 billion yuan. It was the largest in the total economic loss, explaining approximately 72.64 % of the total economic loss. The result was different from that of Chen et al. (2010), who determined that the economic loss caused by premature death was the largest.

2. The highest limit scenario

Table 3 showed there were 178,776 premature deaths caused by PM_10_ and SO_2_. There were 646,282 hospitalizations caused by respiratory disease, 12.66 million emergencies, more than 3090 million restriction days and 1.23 million lower respiratory tract infections and childhood asthma caused by PM_10_. Similarly, the economic loss of the public health effect caused by SO_2_ was also less than that of PM_10_.

**Table 3:** The total highest economic loss of the public health effect caused by the air pollutant in 74 cities

	The coefficient of the dose–response relation	Cases	the loss value estimation (yuan, the mean price from January 2015 to June 2015)	economic loss (million yuan)
PM_10_’s public health effect				
Premature death	2.6	140,028	528,370.2	73,986.62
Hospitalization caused by respiratory disease	12	646,282	2500.5	1616.03
Emergency	235	12,656,359	202.8	2566.71
The number of restriction days	57,500	3,096,768,683	20	61,935.37
Lower respiratory tract infections and childhood asthma	23	1,238,707	114.2	141.46
Asthma	2608	140,458,656	3.57	501.44
Chronic bronchitis	61	3,285,268	68,545.33	225,189.78
Respiratory symptoms	183,000	9,855,802,940	5.28	52,038.64
The total economic loss of the public health effect caused by PM_10_	417,976.05			
SO_2_’s public health effect				
Premature death	2.6	38,748	528,370.2	20,473.29
Chest discomfort	10,000	149,030,000	8.85	1318.92
Lower respiratory tract infections and childhood asthma	5	74,515	8.85	0.66
The total economic loss of the public health effect caused by SO_2_	21,792.86			
The total economic loss of the public health effect	439,768.91			
Economic loss related to public health effects per capita	848 yuan			
The GDP in 74 cities	18,987,854			
The total economic loss of the public health effect accounts for the GDP	2.32 %			

The total economic loss of the public health effect caused by the air pollutant in 74 cities was approximately 439.8 billion yuan in the highest limit scenario, representing approximately 2.32 % of the GDP in 74 cities, a slight difference from the result of Wei et al. (2012). The total highest economic loss of the public health effect caused by PM_10_ was 418 billion yuan, explaining approximately 95.04 % of the total economic loss, which was the major economic loss and was also consistent with the result of Zhang (2012). The economic loss caused by chronic bronchitis was also the largest in the total economic loss, approximately 225.2 billion yuan, explaining approximately 51.24 %. The result was also different from that of Chen et al. (2010).

### The economic loss caused by effect on the public health effect in the major cities

To further understand the health-related economic loss caused by the air pollutant, this paper estimated the health effects of air pollutants and the economic loss in the major cities from January 2015 to June 2015.

1. The lowest limit scenario

**Fig. 3:**
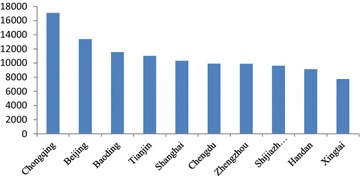
The 10 cities with the highest economic loss (million yuan)

From Fig. 3, Chongqing’s public health economic loss was 17 billion yuan, ranking the first among the 74 cities, followed by Beijing, Baoding, and Tianjin. From the regional perspective, there were 4 municipalities and 4 cities in Hebei Province in the top 10 cities with the highest economic loss. There were 7 cities in North China excluding Chongqing, Shanghai and Chengdu in the top 10 cities with the highest economic loss.

**Fig. 4:**
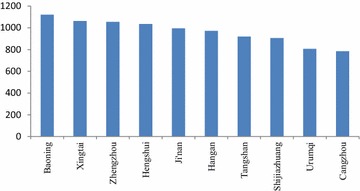
The 10 cities with the highest health-related economic loss per capita (yuan/per person)

As shown in Fig. 4, there were 7 cities in Hebei Province in the top 10 with the highest economic loss. Zhengzhou and Ji’nan also ranked in the top 10 cities due to their poor air quality. Urumqi’s health-related economic loss was not high; however, because of its low population, the resulting health economic loss per capita was higher.

**Fig. 5:**
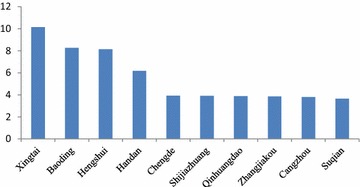
The 10 cities with the highest proportion of health-relate economic loss accounting for GDP (%)

Figure 5 shows that the proportion of the health-related economic loss accounting for GDP in Xingtai was the highest, which was 10.15 %. In the 10 cities with the highest proportion of health-related economic loss accounting for GDP, there were 9 cities in the Hebei province. Suqian in Jiangsu province had a higher health-related economic loss and a lower GDP, ranking the tenth; therefore, the proportion of its health-related economic loss accounting for GDP was higher, ranking in the top 10. The economic losses of the public health effect of 4 municipalities ranked in the top 10. Although their GDPs were higher, the proportions of their health-related economic loss accounting for GDP were relatively lower, which did not rank in the top 10.

2. The highest limit scenario

**Fig. 6:**
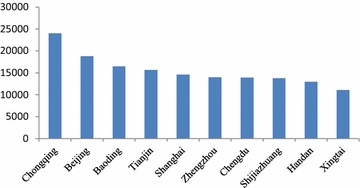
The 10 cities with the highest economic loss (million yuan)

In the highest limit scenario, the cities ranking in the top 10 with the highest economic loss in Fig. 6 were the same as those in the lowest limit scenario in Fig. 3. However, the rankings of Zhengzhou and Chengdu were different in Figs. 4 and 3.

**Fig. 7:**
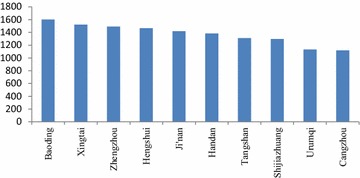
The 10 cities with the highest health-related economic loss per capita (yuan/per person)

The cities ranking in the top 10 with the highest economic loss per capita in Fig. 5 in the highest limit scenario were the same as in the lowest limit scenario in Fig. 4. However, as shown in the Fig. 7 in the following, the economic loss per capita in the top 10 cities in the highest limit scenario was higher than that in the lowest limit scenario. For example, the economic loss per capita in Baoding was 1599.9 yuan/per person, higher by 477.2 yuan than that in the lowest limit scenario.

**Fig. 8:**
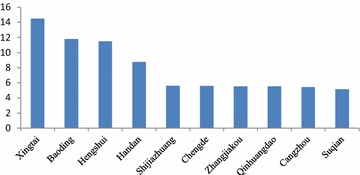
The 10 cities with the highest proportion of the health-related economic loss accounting for GDP (%)

The 10 cities with the highest proportion of health-related economic loss accounting for GDP in Fig. 8 in the highest limit scenario were the same as in the lowest limit scenario in Fig. 3. However, the rankings of Shijiazhuang, Chengde, Zhengjiukou and Qinhuangdao and the proportions of the health-related economic loss accounting for GDP were different in Figs. 8 and 5.

## Conclusions and policy implications

Based on the dose–response relationship and the willingness to pay method, this paper evaluated the health-related economic loss caused by air pollution in China’s 74 cities using the latest available data regarding to PM_10_ and SO_2_ from January 2015 to June 2015, by establishing lowest and highest limit scenarios. The conclusions and policy implications are as follows.

### Conclusions

1. For the whole 74 cities

The health-related economic loss caused by PM_10_ was larger than that caused by SO_2_ in the lowest and highest limit scenarios, and the economic loss associated with chronic bronchitis caused by PM_10_ was the largest in all the losses. Thus, PM_10_ has become the main air pollutant in 74 cities, and it is necessary to focus on the issue of chronic bronchitis caused by PM_10_.

2. For the major cities

In the lowest and highest limit scenarios, the health-related economic loss in Chongqing, Beijing, Baoding, Tianjin and other major cities was larger than in other cities. The health-related economic loss per capita in Baoding, Xingtai and Zhengzhou was higher than in other cities. Regarding to the proportion of the health-related economic loss accounting for GDP, there were 9 cities in the Hebei Province included in the top 10 cities with the highest loss. It was evident that the air pollution was serious in North China, particularly in the Hebei Province, except in Shanghai in East China and in Chongqing and Chengdu in the southwest of China.

### Policy implications

1. According to the results of this paper, Hebei Province is a typical polluted area. Energy consumption structure in Hebei Province is mainly composed of coal resource. Main pollutants from coal combustion are PM_10_ and SO_2_, etc. Thus, to reduce air pollution, the coal-based energy structure needs to be improved, and new energies and advanced production processes need to be utilized. Furthermore, strengthening the development of technology and equipment; improving combustion technology, combustion devices and the fuel utilization rate; and reducing the additional pollutants generated by fuel burning will help reduce air pollution.
2. Pollutant concentration is an important factor affecting the loss of health effects. The government should adopt the emissions trading system, limit the pollutant discharges of enterprises, grasp the influence of the enterprises on environmental pollution, strengthen the management of enterprises, and develop a number of administrative regulations conducive to environmental protection to ensure the implementation of environmental protection measures.
3. From the calculation results, the urban population is also an important factor affecting the loss of health effects. The government should reasonably control the urban population and improve people’s awareness of environmental protection. Accelerating the transfer of industries and upgrading may reduce the non-household population in order to reduce the side-effects caused by population growth and improve the urban environment and public health.

Here the paper uses the data from January to June 2015 to calculate the health-related economic loss in 74 cities. If the paper uses a long term data, for example, which covers from January to December 2015, there may be a larger health-related economic loss (assuming the population in 74 cities is constant). However, the health-related economic loss from January to December 2015 may not be twice as much as that from January to June 2015. As it can been seen from Figs. 1 and 2, the monthly mean concentration data of SO_2_ and PM_10_ from January to June 2015 appeared a downward trend. In the future, data (if complete and available) can be combined using a geographic information system and other new tools to determine the economic loss of caused by the effects of air pollution on public health in the typical resource-based regions or cities and to provide references for environmental management and sustainable development.
